# Efficacy of Statin Monotherapy or in Combination With Coenzyme A Capsule in Patients With Metabolic Syndrome and Mixed Dyslipidemia

**DOI:** 10.14740/jocmr2124w

**Published:** 2015-04-08

**Authors:** Jiangtao Lai, Bifeng Wu, Tianming Xuan, Shudong Xia, Zhong Liu, Junzhu Chen

**Affiliations:** a Department of Cardiology, First Affiliated Hospital, College of Medicine, Zhejiang University, Hangzhou, China

**Keywords:** Coenzyme A, Hypertriglyceridemia, Dyslipidemia, Metabolic syndrome, Combination therapy

## Abstract

**Background:**

Patients with metabolic syndrome are at increased risk for cardiovascular disease. Combination lipid-lowering therapy is often needed in patients with metabolic syndrome and mixed dyslipidemia. The aim of this study was to compare the effect of statin combined with a new hypolipidemic agent, coenzyme A (CoA) with moderate-dose statin monotherapy in subjects with metabolic syndrome and mixed dyslipidemia by evaluating data from a subgroup of patients with metabolic syndrome and mixed dyslipidemia from a previously conducted randomized study.

**Methods:**

In the present *post hoc* analysis, 212 patients were included, receiving statin monotherapy (n = 94) or statin combined with CoA 400 U/day (n = 118) for 8 weeks. The lipoprotein profile was determined at baseline and week 8 visits. Attainment of low-density lipoprotein-cholesterol (LDL-C) < 100 mg/dL, non-high-density lipoprotein-cholesterol (HDL-C) < 130 mg/dL, and the combined goal of these two parameters was also evaluated.

**Results:**

The mean percent change was more prominent with CoA plus statin compared with placebo plus statin in triglyceride (TG) (-32.5% vs. -8.7%, respectively; P = 0.0002), total cholesterol (-9.6% vs. -3.6%, P = 0.013), LDL-C (-7.5% vs. 2.1%, P = 0.033), and non-HDL-C (-14.3% vs. -6.4%, P = 0.011). Treatment with CoA plus statin resulted in larger percentages of participants attaining lipid goals for LDL-C (70.3% vs. 56.4%, P = 0.044), non-HDL-C (60.2% vs. 45.7%, P = 0.039), and the combined goal of LDL-C and non-HDL-C (57.6% vs. 42.6%, P = 0.038) than statin monotherapy.

**Conclusion:**

These results demonstrate that CoA plus statin therapy was more effective in improving lipoprotein parameters than statin alone in patients with metabolic syndrome and mixed hyperlipidemia.

## Introduction

Metabolic syndrome has become increasingly common in the world. Patients with metabolic syndrome often have raised triglycerides (TG), reduced high-density lipoprotein-cholesterol (HDL-C) and sometimes raised low-density lipoprotein-cholesterol (LDL-C) levels [1-3]. Although the definition of metabolic syndrome varies according to ethnicity, metabolic syndrome enhances the risk for cardiovascular disease and pharmacotherapies of the metabolic risk factors are often needed [4, 5].

Current guidelines recommend that patients with metabolic syndrome achieve a target LDL-C < 100 mg/dL as the primary goal of therapy and a target non-HDL-C < 130 mg/dL as the secondary goal of therapy if elevated TG is coexisting [5]. Although lifestyle intervention is critical and may be the initial treatment for patients with metabolic syndrome and dyslipidemia, pharmacological modification is often likely required to achieve lipid goals [6]. Statins are often chose for the initial therapy; however, even in maximally tolerable doses, it often fails to attain desirable lipid goals beyond LDL-C, and therapeutic regimen combining a statin with another hypolipidemic drug may be necessary [7].

Fibrates are often used in combination therapy with a statin in treating mixed dyslipidemia. However, safety issues, especially drug-induced hepatotoxicity, myositis and rhabdomyolysis, are also considered [8, 9]. Coenzyme A (CoA) was a new lipid-lowering agent functioning as an acyl group carrier and involving in the oxidation and catabolism of fatty acids [10, 11]. Animal and clinical studies have shown its normalizing activity on plasma lipids and good tolerability in the treatment of dyslipidemia [12-14].

In a previous study, we randomized 304 patients with mixed dyslipidemia to either moderate-dose statin monotherapy (S group) or statin with CoA 400 U/day (SC group) [14]. The SC combination was better at decreasing TG, total cholesterol (TC), LDL-C and non-HDL-C [14]. The present *post hoc* analysis only included participants with metabolic syndrome from the previous study [14]. We evaluated the efficacy of statin and CoA combination regimen on the changes in the levels and patterns of lipoproteins in patients with metabolic syndrome and mixed dyslipidemia.

## Material and Methods

As a *post hoc* analysis, the present work includes subjects with metabolic syndrome from a randomized, placebo-controlled, phase 3 study that compared the effect and safety of statin plus CoA 400 U combination therapy with moderate dose of statin monotherapy in subjects with mixed dyslipidemia (NCT01928342) [14]. As previously described, men and non-pregnant women who had been receiving moderate dose of a statin (pitavastatin 4 mg/day, rosuvastatin 10 mg/day, atorvastatin 20 mg/day, pravastatin 40 mg/day, lovastatin 40 mg/day, simvastatin 40 mg/day, or fluvastatin 80 mg/day) for the control of raised LDL-C level for at least 8 weeks before screening and had a fasting TG level at 200 - 620 mg/dL on two consecutive visits were recruited [14].

Exclusion criteria were: 1) liver disease with elevations of alanine (ALT) and/or aspartate aminotransferase (AST) > 2 times upper limit of normal (ULN), 2) renal dysfunction as defined by serum creatinine levels ≥ 2.0 mg/dL, 3) poorly controlled hypertension (resting systolic blood pressure ≥ 180 mm Hg and/or diastolic blood pressure ≥ 110 mm Hg at two consecutive visits), 4) unexplained serum creatine phosphokinase (CPK) > 2 times ULN, 5) pregnancy, 6) breast-feeding, 7) women of childbearing age not using any contraception method, 8) established cardiovascular disease, 9) hypothyroidism, 10) heart failure with left ventricular ejection fraction < 30%, and 11) history of receiving therapies with other non-statin hypolipidemic treatment (e.g. fibrates, niacin, or fish oils) during the last 2 months before study entry.

In the present work, only subjects with metabolic syndrome were included. According to the Chinese Guidelines on Prevention and Treatment of Dyslipidemia in Adults, and National Cholesterol Education Program Adult Treatment Panel III (NCEP-ATP III) Criteria for Asian Americans, metabolic syndrome was identified by the diagnostic criteria of abdominal obesity defined by waist circumference ≥ 90 cm for men and ≥ 85 cm for women, TG ≥ 150 mg/dL, HDL-C < 40 mg/dL for men and < 50 mg/dL for women, fasting glucose ≥ 100 mg/dL and blood pressure ≥ 130/85 mm Hg [5, 15]. The diagnosis of metabolic syndrome was identified by the presence of three or more of these components [5, 15]. The study randomized patients at 10 sites in Mainland China. All subjects provided their written informed consent. The study protocol was reviewed and approved by the institutional ethics committee.

At baseline and week 8 visits, blood pressure was measured using a mercury sphygmomanometer. Body weight and height measurements were also performed. Body mass index (BMI) was calculated by body weight/height^2^. Waist circumference was measured in the narrowest part between the lower rib and the top of the hip. Patients were given individualized therapeutic lifestyle changes and dietary instructions, according to the NCEP-ATP III guidelines [5]. During the study period, all participants were asked to visit the clinic monthly for diet compliance assessment.

Patients were randomized to receive moderate-dose statin (the same open-label statin treatment as before with the dosage remaining stable) plus placebo group and moderate-dose statin plus CoA 400 U/day group at the randomization visit. Medication compliance was assessed at week 4 and 8 using a pill count.

Blood samples were obtained for lipoprotein profile and clinical chemistry (including glucose, ALT, AST, serum creatinine, and CPK) analyses at baseline, weeks 4 and 8 after fasting for at least 12 h. These analyses were performed in a central laboratory. Blood samples were mailed to the central laboratory located in First Affiliated Hospital, College of Medicine, Zhejiang University within 48 h in insulated container. Serum lipids and clinical chemistry were analyzed on a Hitachi 7600-210 analyzer (Hitachi High-Technologies, Tokyo, Japan). Pregnancy testing was assessed for women of childbearing potential (chemiluminometric immunoassay).

### Statistical analysis

Data were collected for subjects with a diagnosis of metabolic syndrome at randomization. Of a total of 304 subjects randomized, 212 (69.7%) had metabolic syndrome.

The last observation carried forward method was adopted to impute missing post-baseline values for participants who discontinued the study after randomization. Mean percent changes from baseline to follow-up were assessed by an ANOVA with the baseline lipid parameter value as a covariate. The percentage and number of patients achieving therapeutic goals of LDL-C < 100 mg/dL, non-HDL-C < 130 mg/dL, and the combined therapeutic goal of these two parameters at the end of treatment were also evaluated. Statistics were calculated using SAS/STAT version 9.13 (SAS Institute, Inc., Cary, NC, USA).

## Results

Two hundred twelve subjects with metabolic syndrome were randomized and treated in the study. Two hundred of 212 completed the whole study. The study populations for efficacy evaluation included 212 patients (94 in the S group and 118 in the SC group), receiving at least one dose of the study drug and had post-baseline efficacy data. Baseline clinical and demographic characteristics were similar between the two groups (Table 1). Overall, approximately 75% of participants were < 65 years old; about 60% of participants weighed over 70 kg. The most often used statins at baseline were atorvastatin (64.6%) and simvastatin (18.4%). There were no statistically significant differences between the two groups in the frequency of statins used.

**Table 1:** Demographics and Baseline Characteristics

Characteristic	Statin + CoA (n = 118)	Statin + placebo (n = 94)	P value
Age (years)	55.6 ± 11.6	53.6 ± 13.1	0.25
Male gender (n, %)	65 (55.1)	59 (62.8)	0.27
Current smokers (n, %)	38 (32.2)	36 (38.3)	0.39
BMI (kg/m^2^)	26.0 ± 2.7	25.8 ± 2.7	0.52
Waist circumference (cm)	91.4 ± 10.6	90.9 ± 10.1	0.72
SBP (mm Hg)	134.8 ± 12.5	131.4 ± 14.5	0.07
DBP (mm Hg)	81.6 ± 9.7	81.6 ± 8.7	0.96
Hypertension (n, %)	56 (47.5)	40 (42.6)	0.49
Diabetes mellitus (n, %)	21 (17.8)	23 (24.5)	0.24
ACEIs/ARBs (n, %)	35 (29.7)	24 (25.5)	0.54
β-blockers (n, %)	25 (21.2)	17 (18.1)	0.61
Calcium channel blockers (n, %)	34 (28.8)	18 (19.1)	0.11
Diuretics (%, n)	7 (5.9)	1 (1.1)	0.079
Platelet aggregation inhibitors (n, %)	18 (15.3)	14 (14.9)	1.00
Statins			0.70
Atorvastatin (n, %)	74 (62.7)	63 (67.0)	
Rosuvastatin (n, %)	12 (10.2)	5 (5.3)	
Simvastatin (n, %)	22 (18.6)	17 (18.1)	
Fluvastatin (n, %)	9 (7.6)	7 (7.4)	
Lovastatin (n, %)	1 (0.8)	0 (0.0)	
MetS components (n)	3 (3 - 5)	3 (3 - 5)	0.37
Waist criterion (n, %)	79 (66.9)	63 (67.0)	1.00
FPG criterion (n, %)	82 (69.5)	68 (72.3)	0.76
TG criterion (n, %)	118 (100.0)	94 (100.0)	1.00
HDL-C criterion (n, %)	41 (34.7)	43 (45.7)	0.12
BP criterion (n, %)	93 (78.8)	69 (73.4)	0.42

CoA: coenzyme A; BMI: body mass index; SBP: systolic blood pressure; DBP: diastolic blood pressure; ACEI: angiotensin-converting enzyme inhibitor; ARB: angiotensin receptor blocker; MetS: metabolic syndrome; FPG: fasting plasma glucose; BP: blood pressure.

The presence of metabolic syndrome components was well-balanced between the two groups at baseline. About 70% of patients in each group had increased blood pressure, and all had elevated TG levels. After treatment for 8 weeks, the percentage of patients who met the criteria of metabolic syndrome was significantly reduced in both groups. The proportion of participants who fulfilled the diagnostic criteria of metabolic syndrome was significantly lower in the SC group when compared with S group (80/118 (67.8%) vs. 76/94 (80.9%) respectively, P = 0.041). This was due to a larger reduction in the proportion of patients who met the diagnostic criteria of elevated TG level in this group.

**Figure 1:**
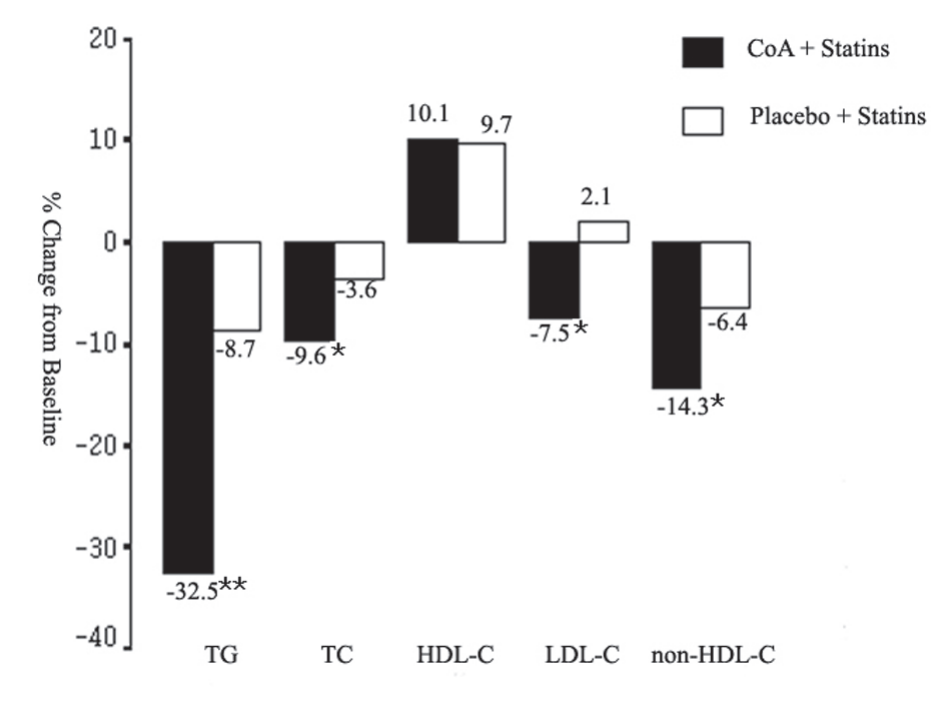
Mean percent change in triglyceride (TG), total cholesterol (TC), high-density lipoprotein cholesterol (HDL-C), low-density lipoprotein cholesterol (LDL-C), and non-HDL-C from baseline to the end of study. CoA: coenzyme A. *P < 0.05; **P < 0.01.

**Figure 2:**
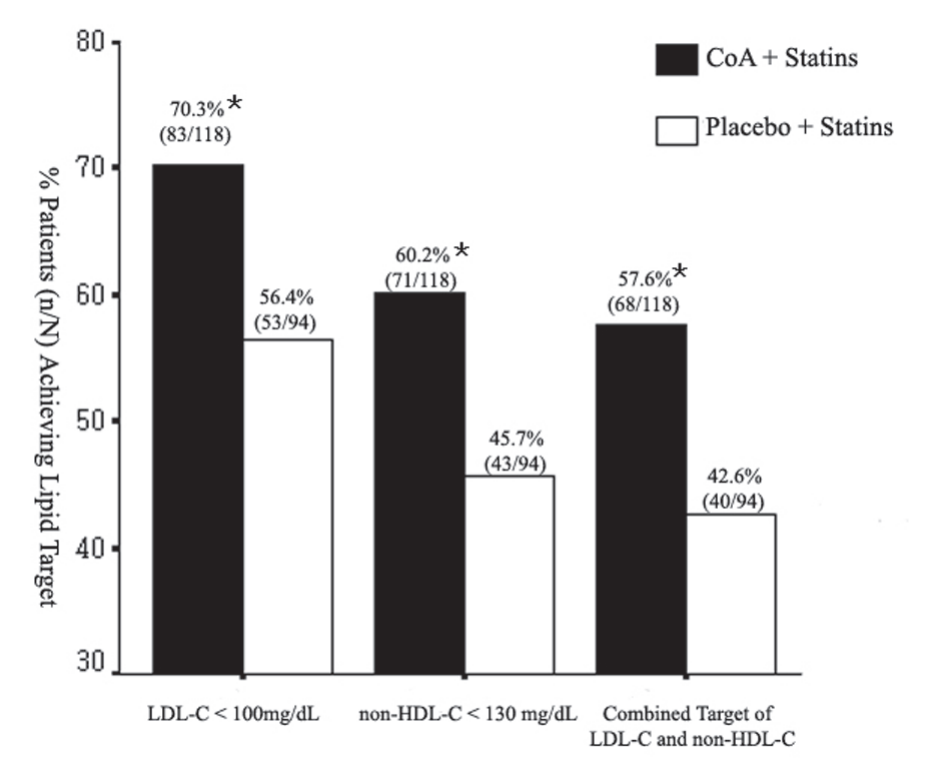
Rates of achievement of the NCEP ATPIII therapeutic goals at the end of treatment. *P < 0.05.

The changes in serum lipoprotein levels are presented in Table 2. Statin plus CoA treatment reduced TC, LDL-C, and non-HDL-C levels significantly when compared with baseline (all, P < 0.01). Significant reduction in non-HDL-C level was also noted in S group (P < 0.05). The changes in these lipid levels were larger in the SC group when compared with the S group (P < 0.05, Fig. 1). So, there were more patients attained the LDL-C and non-HDL-C targets in the SC group (70.3 and 60.2% respectively) than in the S group (56.4 and 45.7% respectively) (both, P < 0.05, Fig. 2). The percentage of participants who attained the combined goal of LDL-C and non-HDL-C was significantly larger with statin plus CoA when compared with statin monotherapy (57.6% vs. 42.6%, P = 0.038, Fig. 2). Both therapeutic regimens reduced TG levels significantly (both, P < 0.05). The reduction was significantly larger in the SC compared with the S group (P = 0.0002, Fig. 1). No significant difference was noted in the percent changes of HDL-C levels between the two groups.

**Table 2:** Percentage Change From Baseline to Follow-Up in Levels of Serum Lipoproteins

Variable	Statin + CoA (n = 118)	Statin + placebo (n = 94)	P value
TG			
Baseline mean (mg/dL)	318.8 ± 114.2	305.5 ± 99.2	
Final mean (mg/dL)	215.2 ± 126.6	275.4 ± 172.7	
Mean change, % ± SE (median)	-32.47 ± 28.30 (-36.81)	-8.69 ± 49.12 (-19.66)	0.0002
TC			
Baseline mean (mg/dL)	204.2 ± 42.9	192.2 ± 42.5	
Final mean (mg/dL)	181.7 ± 38.3	183.3 ± 44.9	
Mean change, % ± SE (median)	-9.56 ± 17.85 (-9.86)	-3.58 ± 16.47 (-4.57)	0.013
HDL-C			
Baseline mean (mg/dL)	45.2 ± 12.4	42.2 ± 11.6	
Final mean (mg/dL)	48.3 ± 13.9	45.6 ± 14.7	
Mean change, % ± SE (median)	10.07 ± 30.08 (5.93)	9.73 ± 27.12 (6.49)	0.93
LDL-C			
Baseline mean (mg/dL)	111.0 ± 35.6	103.2 ± 35.2	
Final mean (mg/dL)	99.4 ± 36.3	100.9 ± 35.2	
Mean change, % ± SE (median)	-7.48 ± 31.40 (-11.36)	2.08 ± 33.09 (-2.84)	0.033
Non-HDL-C			
Baseline mean (mg/dL)	159.3 ± 39.4	150.0 ± 41.0	
Final mean (mg/dL)	133.4 ± 37.5	137.7 ± 43.3	
Mean change, % ± SE (median)	-14.31 ± 22.20 (-15.84)	-6.38 ± 22.40 (-7.38)	0.011

CoA: coenzyme A; TG: triglyceride; TC: total cholesterol; HDL-C: high-density lipoprotein cholesterol; LDL-C: low-density lipoprotein cholesterol.

The safety profile of these two therapeutic regimens was similar. No significant difference was found between the two groups in the proportion of participants who experienced adverse events (Table 3). There were no symptoms leading to treatment discontinuation (e.g. myalgias, gastrointestinal symptoms) or clinically relevant increase of creatine kinase (> 5 × ULN) or AST/ALT (> 3 × ULN) in any group. No more than 3.2% of patients presented mild elevations of creatine kinase < 3 × ULN in each group (Table 3).

**Table 3:** Incidence of Adverse Events

Variable	Total (n = 212)	Statin + CoA (n = 118)	Statin + placebo (n = 94)
Any AE	15	10	5
Patients who experienced any AE	15	10	5
Most common AEs			
Abdominal distention	1	1	0
Nausea	1	1	0
Elevated liver enzymes	1	1	0
Elevated creatine phosphokinase	5	2	3
Upper respiratory tract infection	3	3	0

CoA: coenzyme A; AE: adverse event.

## Discussion

This work is a *post hoc* analysis of a previously published phase 3, double-blinded, randomized, placebo-controlled study that assessed the effect and safety of statin plus CoA combination regimen in patients with mixed dyslipidemia [14]. In the previous study, 304 patients with mixed dyslipidemia were randomized to either moderate dose of statin monotherapy (S) or moderate-dose statin combined with coenzyme A (SC) [14]. The present *post hoc* analysis included only participants with metabolic syndrome. In this analysis, we found that SC reduced TG, LDL-C, and non-HDL-C levels more than statin monotherapy. These findings are consistent with our previous observation of lipid-altering effects in the whole population consisting of participants with mixed dyslipidemia independently of the presence of metabolic syndrome [14].

Because metabolic syndrome presents a string of various cardiometabolic risk factors, it is the main goal of hypolipidemic treatment targeting LDL-C and non-HDL-C levels (second therapeutic target, if TG > 200 mg/dL) in patients with metabolic syndrome and mixed hyperlipidemia according to the NCEP-ATPIII guidelines [5]. Although statins have been considered the first-line intervention for controlling elevated LDL-C levels, quite a lot of patients with high risk of cardiovascular disease cannot achieve optimal lipid goals and adding other hypolipidemic agents may provide additional benefits in optimizing the serum lipoprotein profile [16]. The achievement of LDL-C and/or non-HDL-C targets has already been assessed in lots of clinical studies with statin monotherapy or combinating with other hypolipidemic drugs [17-21]. It was reported that 24-92% of patients treated with different doses of statins monotherapy or combination therapy achieved LDL-C and/or non-HDL-C goals [17-21]. In this analysis, significantly greater percent changes in LDL-C, TG, and non-HDL-C with SC were noted in patients with metabolic syndrome and mixed dyslipidemia, when compared with statin monotherapy. Meanwhile, SC showed its better efficacy in attaining LDL-C, non-HDL-C targets and the combined goal of these two lipoprotein parameters than statin monotherapy. Although there were differences in duration of therapy and study design, the results of target attainment in these studies are similar to the percentage of participants who attained LDL-C and/or non-HDL-C goals with SC in the present analysis.

Combination therapy with statin plus CoA had generally fine tolerability in the subset of participants with metabolic syndrome, consisting with the safety profile of each monotherapy [13, 14]. No myalgia or rhabdomyolysis was reported in this analysis. Occurrences of elevated liver enzymes were infrequent and no abnormal creatine value was noted in this analysis.

CoA capsule was a newly developed lipid-lowering agent. The mechanism of lipid modifying effects with CoA has not been established thoroughly. As one of the most important compounds in the tricarboxylic acid cycle and cofactors for oxidative and biosynthetic reactions in metabolism, adding CoA may promote fat decomposition and normalize different patterns of lipoproteins [22].

The major limitations for this analysis are attributed to constraint of trial design. The duration of therapy was limited to 8 weeks in this study. Although it was sufficient to assess the efficacy of a lipid-lowering agent, it is necessary to know the long-term therapeutic profile of an anti-hyperlipidemic agent. Considering the safety of subjects, only moderate-dose of statins was used in this study, the full therapeutic effect of this regimen may not be accurately reflected in this study and remains unknown. Further studies with longer follow-up duration and higher dose of statin in combining with CoA will be required to fully establish the efficacy and tolerability of co-administration of CoA and statin.

Although these limitations exist, the present analysis clearly demonstrated the efficacy and safety profile of statin plus CoA combination therapeutic regimen in lipid modifying and in achieving individual and combined lipid goals in patients with metabolic syndrome and mixed dyslipidemia.

### Conclusions

In the present work, the addition of CoA 400 U/day to ongoing moderate dose of statin was effective and safe in providing additional lowering of TG, TC, LDL-C, and non-HDL-C levels and achieving individual and combined lipid targets in patients with metabolic syndrome and mixed hyperlipidemia.

## Abbreviations

ALT: alanine aminotransferase
AST: aspartate aminotransferase
BMI: body mass index
CoA: coenzyme A
CPK: creatine phosphokinase
HDL-C: high-density lipoprotein-cholesterol
LDL-C: low-density lipoprotein-cholesterol
S group: statin monotherapy group
SC group: statin-coenzyme A combination group
TG: triglyceride
TC: total cholesterol
ULN: upper limit of normal
